# 4168 Understanding ECM-Based Drug Resistivity in Breast Cancer

**DOI:** 10.1017/cts.2020.346

**Published:** 2020-07-29

**Authors:** Sarah Libring, Aparna Shinde, Miad Boodaghidizaji, Alexandra Plummer, Arezoo Ardekani, Michael Wendt, Luis Solorio

**Affiliations:** 1 Indiana University School of Medicine

## Abstract

OBJECTIVES/GOALS: Cell-cell (CC) and cell-matrix interactions (CM) are known to affect drug sensitivity of cancer cells, but are not effectively recapitulated using 2D platforms. This research aims to determine how cell and matrix interactions confer drug resistivity in 3 distinct culturing models: 2D (no CM/limited CC), 3D spheroids (CC) and 3D fibronectin (both). METHODS/STUDY POPULATION: We examined four breast cancer cell types. The cells were derived from a nonmetastatic primary tumor (HMLE-E2) or overt bone-metastasis (BM). Transglutaminase 2 (TGM2), a matrix crosslinking protein, is overexpressed in metastatic bone tumors and may play a key role in matrix-conferred drug resistivity. In a gain-of-function model, TGM2 was upregulated in HMLE-E2 cells and compared to shTGM2 knockdown BM cells. Growth rates were analyzed using metabolic activity over 8 days, and drug sensitivity to Neratinib (0-1000 nM) was analyzed via cell titer. To account for the different transport properties of the 3 distinct culture environments, we developed a mathematical model for each condition, allowing us to normalize the drug sensitivity results across models to effectively compare true biological resistivity. RESULTS/ANTICIPATED RESULTS: We observed that increased cellular levels of TGM2 significantly increase the growth rate and drug resistivity of cells on fibronectin matrices. Interestingly, in 2D cultures, TGM2 expression was correlated with higher Neratinib resistivity but did not affect growth rates. In spheroid models without a significant matrix component, that rely solely on cell-cell junctions, high levels of TGM2 were correlated with lower survival rates. Lower levels of TGM2 are correlated with a more epithelial phenotype, and using our mathematical model we have identified significant transport differences between high and low TGM2 spheroids. We theorize that the low TGM2 spheroids have denser packing, which lowers the rate of diffusion and, thus reduces the effective concentration of the drug to the majority of the cells. DISCUSSION/SIGNIFICANCE OF IMPACT: Our studies indicate that the cellular response to drugs can be altered by changes in both transport properties of the tissue and the CM interactions. By systematically investigating the effects of CC interactions and CM interactions, we can use mathematical models to delineate physical means of drug resistivity from a biologically driven resistance.

